# Supplementary material for: Assessment of Functional and Nutritional Status and Skeletal Muscle Mass for the Prognosis of Critically Ill Solid Cancer Patients
Source: Cancers (Basel). 2022 Nov 29;14(23):5870. doi: 10.3390/cancers14235870 (PMC9737490; doi:10.3390/cancers14235870)
Supplement: Supplementary file 1 [file cancers-14-05870-s001.zip › cancers-1995957-supplementary.pdf]

Supplementary

Assessment of Functional and Nutritional Status and Skeletal Muscle Mass for the Prognosis of Critically Ill Solid Cancer Patients

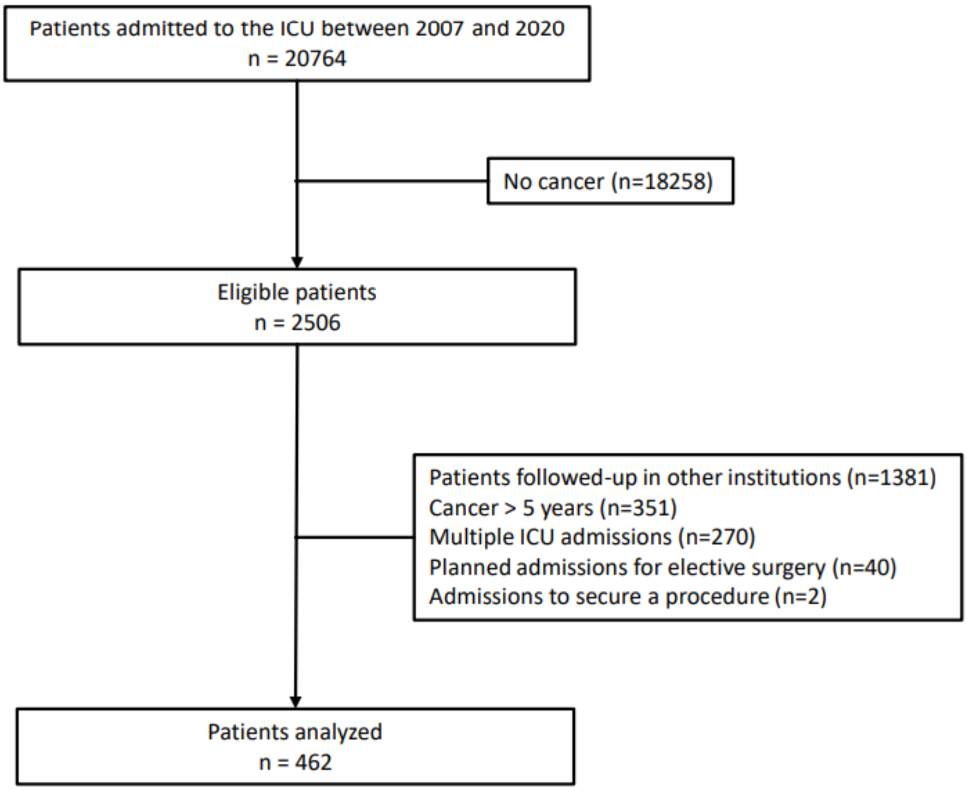

Figure S1. Flow-chart.
